# Predicting hydration layers on surfaces using deep learning†

**DOI:** 10.1039/d1na00253h

**Published:** 2021-05-06

**Authors:** Yashasvi S. Ranawat, Ygor M. Jaques, Adam S. Foster

**Affiliations:** Department of Applied Physics, Aalto University Finland yashasvi.ranawat@aalto.fi ymjaques@gmail.com adam.foster@aalto.fi; WPI Nano Life Science Institute (WPI-NanoLSI), Kanazawa University Kakuma-machi Kanazawa 920-1192 Japan

## Abstract

Characterisation of the nanoscale interface formed between minerals and water is essential to the understanding of natural processes, such as biomineralization, and to develop new technologies where function is dominated by the mineral–water interface. Atomic force microscopy offers the potential to characterize solid–liquid interfaces in high-resolution, with several experimental and theoretical studies offering molecular scale resolution by linking measurements directly to water density on the surface. However, the theoretical techniques used to interpret such results are computationally intensive and development of the approach has been limited by interpretation challenges. In this work, we develop a deep learning architecture to learn the solid–liquid interface of polymorphs of calcium carbonate, allowing for the rapid predictions of density profiles with reasonable accuracy.

## Abstract 2

We present a novel deep-learning technique to predict hydration layer density over surfaces. This will complement atomic force microscopy techniques in characterizing the solid–liquid interfaces.

## Graphical abstract

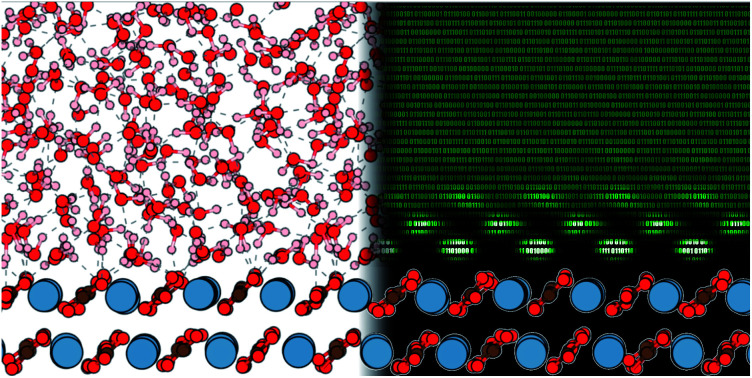

Solid–liquid interfaces are ubiquitous in nanoscale materials' science.^1^ At the atomic scale, liquid molecules are differently adsorbed on the surface, forming complex hydration structures. These atomic scale interactions largely influence macroscopic surface phenomena,^2^ which in turn drive various natural and technological processes.^3^ Atomic force microscopy (AFM)^4^ is being developed to image these critical atomic interactions at high speed—commensurate to the molecular-diffusion time-scales—and atomic-scale resolution.^5–14^ However, the interpretation of the AFM images at such resolutions still remains a massive challenge. The measured force between an AFM tip and the surface hydration layers in the solution comprises tip–surface–solvent interactions and entropic effects, and linking this to the image-contrast mechanism requires an intensive modelling approach for reliable understanding.^15–18^

The demonstration of a direct relationship between experimental AFM force data and water densities opened an easier route to interpret the images through simulations,^19,20^ which were further advanced by inclusion of the influence of the tip's hydration structure in the forces^21^ and an analysis of the role of tip radius.^17^ However, these studies still require detailed molecular dynamics (MD) simulation of water over various estimates of surface structures to describe the hydration structure formed at the mineral–liquid interface – this is computationally expensive and requires complex parameterisation of classical force fields. These technical challenges encumber nanoscale characterisation of solid–liquid interfaces through AFM and is in stark contrast to the breakthroughs in molecular characterization offered by low-temperature functional-tip AFM in ultra-high vacuum.^22,23^ In an effort to provide a rapid and reliable interpretation tool, in this work we develop a machine learning approach that can predict water densities, and hence AFM images, directly from the atomic structure.

In order to benchmark our approach, we focus on ideal and defected surfaces of calcium carbonate (CaCO_3_) polymorphs^25^ (see Fig. 1). The calcite (101̄4) surface has become a benchmark surface for AFM studies in liquids.^12^ Alongside this, a wealth of computational studies of the calcite–water interface have been undertaken, providing a critical background of structural understanding and reliable forces fields necessary for our approach. These studies extensively delineate the calcite (101̄4) surface and water interactions,^26,27^ AFM imaging,^16,28–31^ surface ion dissolution,^32,33^ and effects of point defects and step edges on the hydration layer densities.^14,34–37^ Alongside calcite, we further consider the other most common polymorphs of CaCO_3_, aragonite and vaterite. Aragonite is the second most stable polymorph of CaCO_3_, and plays a vital role in biomineralization.^38^ Surfaces of aragonite show predominantly the (001), (010) and (110) planes.^39^ In these surfaces, the carbonate groups are aligned differently from those in (101̄4) calcite, *cif.*Fig. 1. Experimental works^40,41^ have found clearly distinct patterns in the hydration layer of aragonite (001) surface in comparison to calcite (101̄4). Secondly, of those commonly considered, vaterite is the least thermodynamically stable polymorph of CaCO_3_, and is possibly related to calcite nucleation and dissolution processes.^42^ The vaterite surface structure has been elusive, as only micro and nano-sized crystals are obtained^42^ and large enough areas for reliable experimental characterization are rare – as yet, there are no experimental results on the interface of water with vaterite. Current studies indicate two competing/interspersed structures with space groups *P*3_2_21 and *C*121.^42,43^ We use the *P*3_2_21 structure with the (010) cleavage plane, since this was found to have one of the lowest surface energies.^44,45^ The different morphologies of these polymorphs, as compared to calcite, and their significant applications make them suitable test cases.

**Fig. 1:**
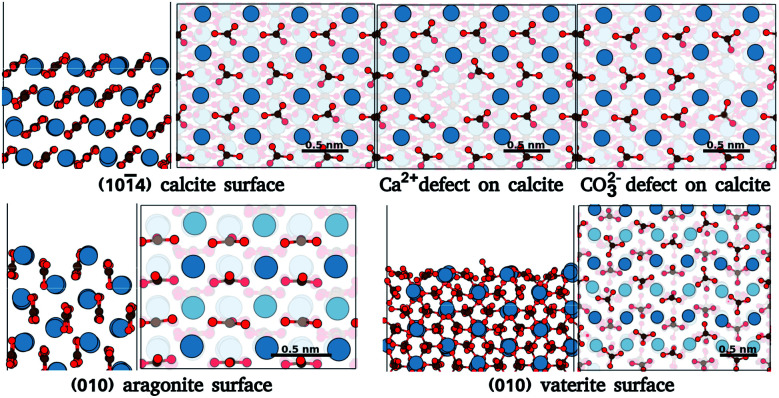
Surface atomic structures of the different polymorphs of calcium carbonate: calcite (101̄4) surface, aragonite (010) surfaces, and vaterite (010) surface. For each case, the slab and the top view of the surface are shown. Each surface figure has two translucent layers added to ease the surface visualisation, the first translucent layer demarcates the top layer of a slab, while the second translucent layer demarcates the depths of atoms in the top layer. The atoms of calcium, oxygen, and carbon are represented by blue, red, and brown spheres. (The atomic structure is imaged using VESTA^24^).

Our methodological approach builds upon the general philosophy of using machine learning to handle data analysis challenges in Scanning Probe Microscopy (SPM)^46–49^ and the specific use of deep learning Convolutional Neural Networks (CNN)^50^ to recognize features in high-resolution SPM images. Recent examples include conditioning of SPM tips,^51^ identification of defects with STM^52,53^ and nanostructures with AFM,^54^ and making molecular structure predictions from AFM images.^55^ However, to the best of our knowledge, no earlier studies have applied machine learning to SPM at solid–liquid interfaces. We also note that despite sharing some common machine learning algorithms, in each case mentioned, the training data and machine learning architecture are designed for the system being studied, so that they can be effectively considered entirely different computational models from the present applications in SPM.

In this work, we aim to derive a robust computational tool that could predict the hydration layers over surfaces (see Fig. 2), and ease the rapid discovery of possible surface structure from candidate surfaces. This can provide experiments with unprecedented on-the-fly AFM analysis in liquids over CaCO_3_ surfaces. Further, we progressively include data from molecular dynamics (MD) simulations of water with calcium carbonate polymorphs into the machine learning training to determine the general applicability of these deep-learning techniques to hydration structure prediction. This opens the door to a method for rapid and reliable interpretation of any system imaged by AFM in liquids in the future.

**Fig. 2:**
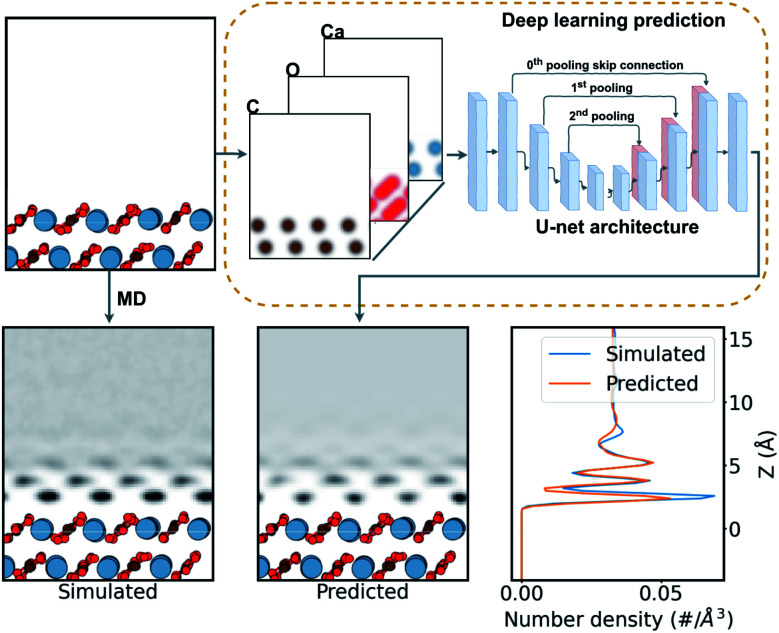
Schematic of the prediction process of water density over a calcite surface. The surface structure is firstly expressed as a three-channel density of Gaussian-smeared carbon, oxygen, and calcium atoms. Then this density is fed into the neural network, shown as a schematic of the U-net network (see ESI† for more details). The network predicts a single-channel density of water over the surface. This is compared with the MD-simulated water density as a target.

To generate a neural network training set for each surface considered, we calculated the water density over the surfaces using the LAMMPS MD code^56^ (for a more detail discussion see the ESI†). We utilized the inter-atomic potentials from Raiteri *et al.*,^33^ a well established potential for carbonate minerals in the context of AFM imaging in liquids^14,16,34^ that implements the SPC/Fw flexible model for water.^57^

We trained our models on the (101̄4) calcite dataset, and the combined (101̄4) calcite and (010) aragonite dataset, to gauge the generality of the models trained. To create the large database, which includes a wide variety of local chemical environments—crucial for an effective learning—we generated a wide variety of the (101̄4) calcite and the (010) aragonite surfaces by removing permutations of Ca^2+^ and CO_3_^2−^ pairs from the surface of each mineral, always retaining net charge zero in the system. We generated 26 784 cases of the calcite dataset and 51 336 cases of the calcite + aragonite dataset. Each data pair, of input and target, comprises three-channels of the atomic-density of carbon, oxygen, and calcium from the surface as input, and water over the surface as the target, as seen in Fig. 2. These densities are given in a 10 × 10 × 20 Å^3^ volume (see ESI† for data extraction method). For training, the dataset was split into 70% training, 20% validation, and 10% testing cases, approximately.

The task of simulating the hydration structures over a given surface can be categorised as an image-to-image problem^58^ in computer vision (see Fig. 2), specifically in three dimensions. Machine learning models tackling such image-to-image problems have originated from the introduction of encoder–decoder models in machine translation.^59^ These models distill the input into a condensed latent-space feature-vector using an encoder, that retains only the higher-level semantic information. This information is then translated by the decoder into the output. The U-net CNN architecture^60^ further enhances such encoder–decoder models with their introduction of skip connections, connecting layers in the encoder to the decoder. This mitigates any loss of spacial information during the encoding in image-to-image applications. Recently, many such image-to-image deep learning methods have found their applications in image segmentation,^58,60,61^ high resolution image synthesis,^62^ image synthesis from semantic labels,^62,63^ and style transfer,^64,65^ among others. Further, the introduction of the attention mechanism^66^ in ML models has seen improvements in machine translation. The success is attributed to their ability to draw the global spacial dependencies in the inputs.^66^ A transformer architecture,^67^ an attention mechanism variant, has also been successfully extended to image-to-image problems.^61,68,69^

Here we trained the data with the U-net architecture, with three pooling scales and their corresponding skip connections,^60^ as seen in Fig. 2. We also applied a soft self-attention mechanism^61^ to the skip connections to derive an attention variant of the U-net in order to provide more detail on what the network is actually focusing on (attention U-net – for a more detailed discussion see ESI†).

The surfaces of calcite with CO_3_^2−^ and Ca^2+^ defects and pristine surfaces of (010) aragonite, (001) aragonite, and (010) vaterite are used to test the neural network predictions. These surfaces are unseen by the networks during training, and thus they represent reasonable test cases. We use mean absolute error (MAE)—L1 loss function—to derive a figure-of-merit to compare the predictions in various cases. We also plot and qualitatively compare the simulated and predicted densities in three ways: the 1D mean density along the *z* direction, the 2D mean density from in the *xz* plane, and finally, *xy* slices corresponding to the peaks in water density along the *z* direction. Moreover, we analyse the attention with respect to atom positions in the (010) aragonite surface case to gain an insight into the evaluations exercised by the network during the predictions.

The MAE of prediction of the U-net and the attention U-net is shown in Table 1. In general, these predictions took less than a second on a standard desktop computer. It is seen from the table that both the simple and attention U-net networks show low errors on their respective test sets. The prediction errors for simple U-net are lower than the attention variant, albeit with minor differences. Naturally, the absolute errors by networks are higher on subsequent test surfaces than that in their test datasets, including for defected systems (where the errors are calculated within 3 Å of the defect site). In general, the overall prediction errors are low, giving us confidence in the reliability of the method. To further generalise the networks, they are also trained with the calcite + (010) aragonite dataset. With the new morphologies incorporated in the network training, the errors of the network are lower on the test dataset. The predictions over the unseen defects and surfaces are similar to that of the networks trained with just the calcite dataset, albeit with slightly higher errors. Only a small change in prediction error with the additional inclusion of distinct surfaces implies generality of the trained networks to materials beyond calcium carbonate in the future. Since there are no significant benefits to the expanded training set for the examples we consider in detail, we focus on the network trained on the calcite dataset alone (using U-net unless otherwise mentioned).

**Table 1:** Comparison of U-net and attention U-net, trained on calcite data (c) and calcite + aragonite data (c + a). MAE of the predictions of the networks for the respective test datasets, pristine (010) aragonite, (001) aragonite, and (010) CO_3_^2−^ terminated vaterite surfaces, and (101̄4) calcite surfaces with CO_3_^2−^ and Ca^2+^ defect is presented

	U-net (c)	Att U-net (c)	U-net (c + a)	Att U-net (c + a)
Respective test set	**1.1015** × **10**^**−3**^	1.1433 × 10^−3^	**8.8640 × 10** ^**−4**^	1.0575 × 10^−3^
CO_3_^2−^ point defect	**2.7288 × 10** ^**−3**^	3.0565 × 10^−3^	**2.8357 × 10** ^**−3**^	3.1721 × 10^−3^
Ca^2+^ point defect	2.6579 × 10^−3^	**2.6496** × **10**^**−3**^	2.9125 × 10^−3^	**2.8362 × 10** ^**−3**^
(010) aragonite	**9.4199** × **10**^**−3**^	1.0148 × 10^−2^	*In training data*	*In training data*
(010) CO_3_^2−^ vaterite	**6.4501 × 10** ^**−3**^	7.4893 × 10^−3^	**6.6732 × 10** ^**−3**^	7.9105 × 10^−3^

In order to confirm that this *quantitative* accuracy translates into a meaningful *qualitative* description of the water density, we now consider each system in detail. Firstly, we look at the case of the CO_3_^2−^ vacancy defect in the (101̄4) calcite surface (Ca^2+^ vacancy is discussed in the ESI†). From Fig. 3, we see that in the 1D plot the position of the peaks are very well predicted, with slight differences in the peak magnitudes. The differences in magnitude can be attributed to the choice of loss function – a network trained with a L1 loss function has been shown to predict blurry images in image-to-image models.^58^ From the 2D slice, it is seen that the network is able to predict the water density in the defect region, below the surface. The network is also able to predict the perturbation in slices further from the surface. The extent is visible up to ≈5 Å from the surface. This corroborates with the previous experimental defect study by Söngen *et al.*^14^ Notwithstanding the stability of the (101̄4) cleavage plane, some extreme neutral defects created in the training process lead to the Ca^2+^ and the CO_3_^2−^ ions to diffuse into the second or the third hydration layers. We suspect these *unexpected* solvated ions improve the generality of the learning process and play a role in the high accuracy of prediction over ionic defects.

**Fig. 3:**
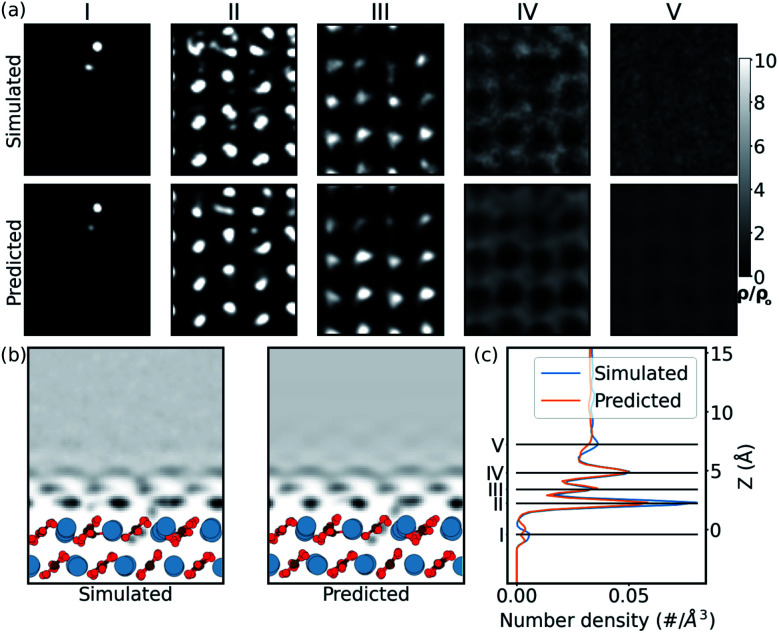
Prediction of hydration layers over a calcite surface with CO_3_^2−^ vacancy using the U-net. (a) Comparison of 2D slices in simulated and predicted water density at *z* heights corresponding to the peaks in the simulated data. The density (*ρ*) is scaled with the bulk water density (*ρ*_o_) for the 2D slices. (b) The mean water density in the 2D *xz* plane, (c) the 1D water density along the *z* direction.

In the case of the prediction over the pristine (010) aragonite surface (the (001) aragonite surface is discussed in ESI†) the errors are larger. We attribute this to the very different morphology of aragonite as compared to the calcite surfaces in the training cases. The resulting hydration density prediction, with simple U-net, over the aragonite surface is compared with the simulated density in Fig. 4. There we can see that the 2D predicted slices at the first and third peak positions, are similar to the simulated data. The predicted slice at second peak is unable to capture detailed features in that position, albeit following the same trends from the simulated data. This is further visible in the mean density along the *z* direction, in the *xz* plane. In the comparison of 1D mean density along the *z* direction, the network predicts all the peak positions remarkably well, but with lower magnitudes, just like the prediction in CO_3_^2−^ defect.

**Fig. 4:**
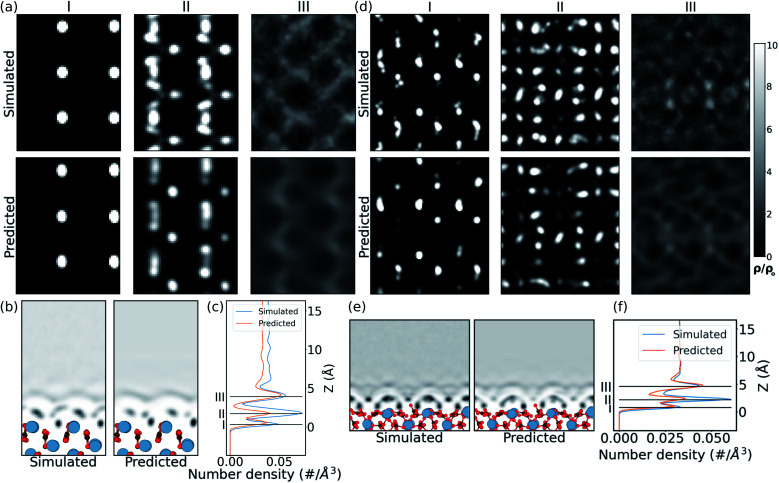
Prediction of (010) aragonite (a–c) and vaterite (d–f) surfaces using the U-net, trained on calcite data. (a and d) 2D slices in simulated and predicted water density at *z* heights corresponding to the peaks in the simulated data. The density (*ρ*) is scaled with the bulk water density (*ρ*_o_) for the 2D slices. (b and e) Comparison of the mean water density in the 2D *xz* plane. (c and f) The 1D water density along the *z* direction.

The approach is also tested on the vaterite surface (see Fig. 4). The prediction errors are found to be lower than in the aragonite case. The hydration density peaks, like the aragonite and CO_3_^2−^ cases, are predicted with lower magnitudes, although at correct heights, *cif.*Fig. 4. The 2D slices in predicted density are visibly similar to that in the simulated case, although some features are somewhat fainter in comparison. We note that should molecularly resolved AFM images of water on vaterite surface be available, our approach could be used to easily compare possible surface structures without any of the associated complexity of modelling thermodynamic stability and/or deriving new force fields.

Finally, in order to try and understand in more detail how the machine learning makes predictions of the density, we now consider results using the Att U-net. The attention values coming from the network prediction over the aragonite surface are shown in Fig. 5. The 1D attention values are plotted along *z* direction at different scales. In the network, these attention values are extracted from the skip connections at three pooling scales: 0^th^ (with no scaling), 1^st^, and 2^nd^, *cif.*Fig. 2. It is seen that the perturbations in the attention values align with the atoms from the surface, especially with the Ca^2+^ ions. It is clear that more attention is given to the ≈5 Å region above the surface, where most water density peaks reside. While this applies to the 0^th^ and 1^st^ pooling scales, the attention at the 2^nd^ scale, with the widest reaching kernel, demarcates the surface with the bulk water density. This reveals that the layers at each scale play different roles during prediction. Additionally, the attention at the 0^th^ pooling scale is weighted with the carbon, oxygen, and calcium densities and visualised at the same isosurface value in Fig. 5. The atom-density-weighted attention shows higher affinity of the network in regions with higher probability of water around the atom. This apparent correlation of these attention values with the elements in the space implies some learning of real-space physics in the network at the layers at every scale. However, in the weighted-attention isovalues of Ca^2+^ the attention is positioned on top regardless of the layer-depth of the atom. This highlights a bias in the network towards predicting water on top of atomic structures, *i.e.* surfaces with surface normal towards *z* direction. We attribute this to all the cases in the training data being surfaces and should be noted when considering generalizing the method to other structures. In particular, nanoclusters are commonly used to depict an AFM tip in simulations and would require further development to be incorporated reliably.

**Fig. 5:**
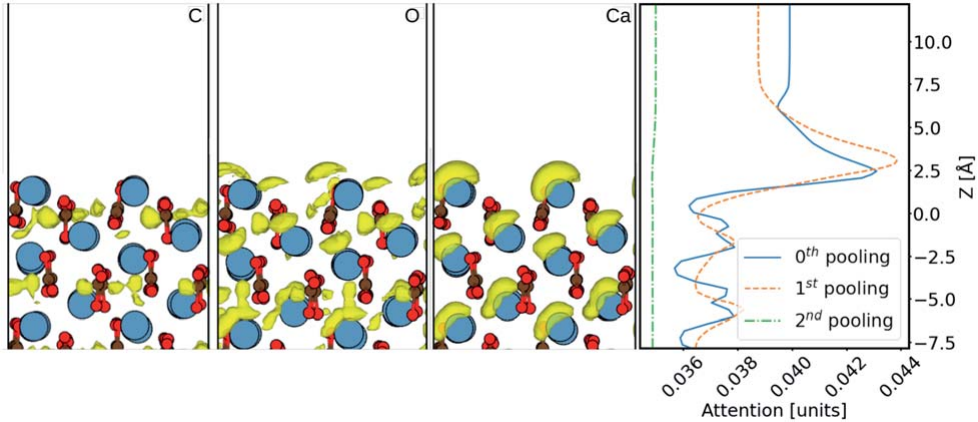
Comparison of attention output at 0^th^ pooling with the atoms of C, O and Ca in the surface, during prediction over the (010) aragonite surface. The first three panels are 2D snapshots of the 3D attention-density iso-surface corresponding to the C, O, and Ca, respectively. The fourth panel has 1D mean densities of the attention output, at different pooling scales.

The simple and the attention variant of the U-net CNN are used to predict the water densities over polymorphs of calcite. The network is shown to learn the water densities, with reasonable peak locations, albeit lower magnitudes near the surface. The generality of the network is established by comparing the loss over surfaces not seen by the network, when trained with calcite data and calcite + aragonite data. The attention mechanism is shown to indicate parallels of the inner layer mechanism with the physical nature of the interactions.

The network makes it possible to predict hydration densities over any surface of calcite, and will help expedite surface characterisation during experiments. Further inclusion of other surface orientations can extend the network to predict water densities around, for example, nanoclusters. It also paves the way for networks that can predict water densities over systems with wider elemental composition than carbon, oxygen, and calcium. This will likely require development of the descriptor used in the training process, taking into account a more detailed description of the local chemical character than offered by the elemental name alone.^70,71^

## Conflicts of interest

There are no conflicts to declare.

## Supplementary Material

NA-003-D1NA00253H-s001
