# Supplementary material for: Predicting hydration layers on surfaces using deep learning
Source: Nanoscale Adv. 2021 May 6;3(12):3447–53. doi: 10.1039/d1na00253h (PMC9419798; doi:10.1039/d1na00253h)
Supplement: NA-003-D1NA00253H-s001 [file NA-003-D1NA00253H-s001.pdf]

Electronic Supplementary Material (ESI) for Nanoscale Advances.
This journal is © The Royal Society of Chemistry 2022

# Supplementary Information: Predicting hydration layers on surfaces using deep learning

Yashasvi S. Ranawat,[a] Ygor M. Jaques,[a] and Adam S. Foster[ab]

## Methods

### Molecular dynamics

We created a nine-layer slab of calcite of dimensions 1.995 × 1.650 × 2.710 nm$^3$, with the (10$\bar{1}$4) surface facing the $z$ direction of the simulation box and using the crystallography data from[1]. The mineral is periodic in $x$ and $y$ directions. The box length in $z$ is 7.430 nm, resulting in a spacing between slabs of 4.720 nm in which 530 water molecules were added. For aragonite, we studied two structures: (010) and (001), creating the slabs from the crystallography data from[2]. The (010) surface consisted of a slab of dimensions 2.297 × 1.984 × 3.115 nm$^3$, with the (010) plane facing the $z$ direction of the simulation box. The total box length in $z$ is 7.599 nm, with the space between slabs being filled with 800 water molecules. The (001) surface consisted of a slab of dimensions 3.008 × 2.403 × 2.366 nm$^3$, with the (001) facing the $z$ direction of the simulation box. The total box length in $z$ is 6.949 nm, with the space between slabs being filled with 1200 water molecules. The vaterite surface was built by using the crystallography data from[3] with space group P$3_2$21. We cleaved the structure at the 010 plane, creating a slab with dimensions 2.516 × 2.154 × 2.608 nm$^3$. The total box length in z is 7.909 nm, with the space between slabs filled by a 1000 water molecules.

For each system, we performed a series of equilibration steps: 100 ps of NVE (constant number of particles, volume, and energy) 500 ps of NVT (constant number of particles, volume, temperature) at 300 K, 1 ns of NPT (constant number of particles, pressure, and temperature) at 300 K and 1 atm of pressure coupling along the $z$ direction; then finally, a production run of 2 ns on NVT, at 300K. The equilibration time is suitable to equilibrate the surface ions and the hydration layers. Moreover, the run time of the production run is long enough to give averaged densities that can be captured by the ML approach. We utilized Nose-Hoover thermostats and barostats with dampening factors of 0.1 ps to control temperature, and of 1 ps to control pressure. The averaged density of water oxygen from the production run is used to denote the water density over the surface. This approach is consistent with the previous works[4–6]. Particle-particle-particle-mesh method[7] was used to calculate the long-range electrostatic interactions.

### Training set

For calcite, we started with a surface of 4×4 $CaCO_3$ units along x and y directions, cif. fig S1, to create the surface defects. The densities of $C_{surface}$, $O_{surface}$, $Ca_{surface}$, and $O_{water}$ were derived during the production run and discretized as 3D-matrix histograms

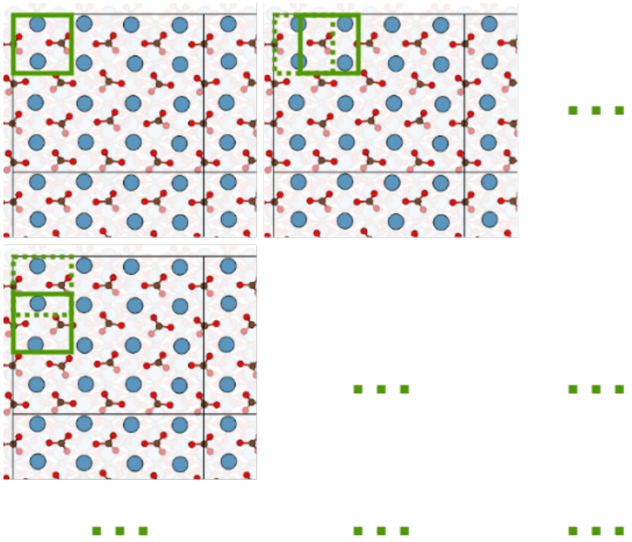

**Fig. S1** Schematic of the process of taking training data from the calcite database. The calcite slab, with marked unit cell, is shown from top, with the box of cropped density. It is ensured that the surface is shared in the subsequent box locations. The atoms of calcium, oxygen, and carbon are represented by blue, red, and brown spheres. (The atomic structure is imaged using VESTA[8])

(voxel size of 0.2 Å). We smeared the densities with a gaussian to extend the effect of the point sized atoms — the $C_{surface}$, $O_{surface}$, and $Ca_{surface}$ — and to reduce the statistical-sampling noises in the water density — the $O_{water}$ — from the trajectory. Next, we split this data-set into volumes of $10 \times 10 \times 20$ Å$^3$, in a way that each block contained the top two layers of the surface and the interfacial $O_{water}$ density, as seen in Fig. S1. We chose this volume size because it minimises GPU memory required during the training, while suitably covering the effect of defects on the hydration layer density. This results in a pre-processed data size of 26,784; consisting of training, validation, and test datasets of sizes 19,280, 4,824, and 2,680, respectively.

Similar to the calcite case, we generated a dataset of 1024 cases comprising defects on the surface of aragonite. Combining it with the preprocessed calcite data, we obtained a final dataset with 51,336 structures; consisting of training, validation, and test datasets of sizes 39,960, 9,240, and 5,136, respectively. We trained the ML model with this combined dataset to estimate its overfitting and to determine its generality.

### Machine learning

The U-Net comprises 3D convolution neural networks chained like the encoder-decoder models[9]. Our network had three scale pooling until the encoded latent space and skip connections, which connect the layers in the encoder to the ones in the de-

[a] *Department of Applied Physics, Aalto University, Finland*
[b] *WPI Nano Life Science Institute (WPI-NanoLSI), Kanazawa University, Kakuma-machi, Kanazawa 920-1192, Japan*

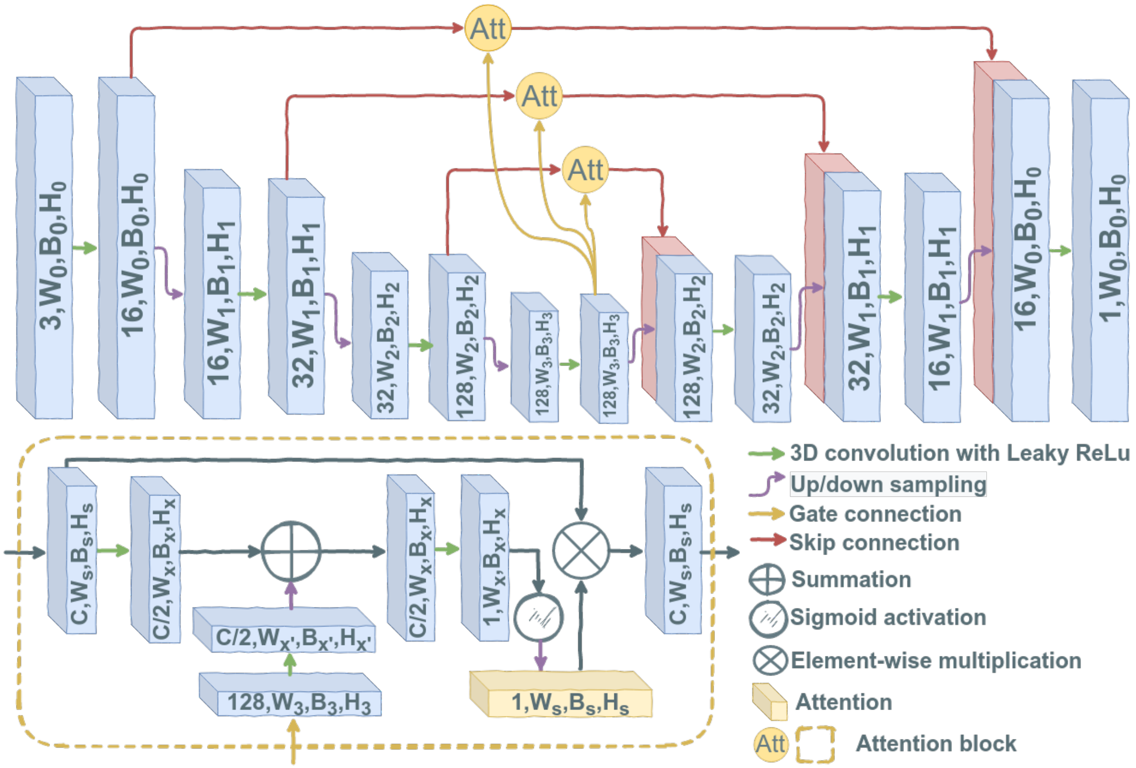


**Fig. S2** Schematics of the attention U-Net. The simple U-net has the same encoder-decoder architecture with skip-connections, but without the attention mechanism. The network starts with the input densities as three channels comprising C, O, and Ca from the surface. Through a series of convolutions and activation filters, along with (attention mapped) skip connections, the output is calculated as one channel of water density.

coder, as seen in fig S2. All the 3D convolution layers, denoted as $\phi(x_l, w)$, with $x_l$ as input at layer $l$ and $w$ as weights, are followed by a leaky ReLU activation layer[10] $\alpha(\phi(x_i, w))$ to induce non-linearity in the model, cif. equation 1. The skip connections, red lines in fig S2, concatenates the output of the layer prior to pooling, during encoding, to the output of the layer up-scaled to the same scale, during decoding. These update rules are as follows:

$$x_l = \alpha_l(\phi_l(x_{l-1}, w_l)) \tag{1}$$

$$x^{cat}_{n-l} = Concatenate(Upscale(x_{n-l}), x_l) \tag{2}$$

$$\begin{aligned}\frac{\delta L(y,\Theta(x_i))}{\delta w_l} &= \frac{\delta L(y,\Theta(x_i))}{\delta x_l}\frac{\delta x_l}{\delta \phi_l(x_{l-1},w_l)}\frac{\delta \phi_l(x_{l-1},w_l)}{\delta w_l} \\ &= \left(\frac{\delta L(y,\Theta(x_i))}{\delta x_{l+1}} w_{l+1}\alpha'_{l+1} + \frac{\delta L(y,\Theta(x_i))}{\delta x^{cat}_{n-l}} w_{n-l}\alpha'_{n-l}\right)\alpha'_l x_l\end{aligned} \tag{3}$$

where $\alpha'_l$ is the differential of the activation layer with input $x_l$. In equation 2 the $x^{cat}_{n-1}$ is the concatenation of the output after up-scaling and output prior to pooling. This allows for a greater significance of the input over the deeper layers, in decoder, which mitigates the loss of the semantics from the input. The equation 3 shows the gradient of the loss function $L$ with respect to the weights $w_l$ of the layer prior to pooling, using back-propagation[11]. Back-propagation uses the chain rule of differentiation to calculate this gradient in terms of the gradient at the next layers; which is calculated in terms of the gradient in the subsequent layers, until the loss value. These gradients are used to update the weights during training. At the layer prior to pooling, this gradient is expressed as a summation of the gradient at the layer after the skip connection, and the gradient at the layer after the pooling. This permits the shallower layers during encoding, to have a significant gradient during training from deeper layers. Thus, less training is required to learn the semantics, due to the skip connections.

We applied a soft self-attention mechanism[12] in the attention variant of the U-Net. The output of the lowest encoded latent-space scale (up-scaled to skip value) is used as the key. This allows for non-local semantic learning in the attention mechanism. The skip value is also used as the query and, hence, the mechanism is called self-attention. The linear transformations applied to the query and the key are voided of spatial information, by using $1 \times 1 \times 1$ kernel convolutions, thus keeping the network small[12]. We derived the attention value using sigmoid activation. This attention is multiplied element-wise to the skip connection, given as:

$$x^{att}_{ijk} = Attention(x_{ijk}, x^{latent}_{ijk})x_{ijk} \tag{4}$$

During training, the weights are updated using ADADELTA scheme[13]. The mean absolute error (MAE) — the L1 loss function

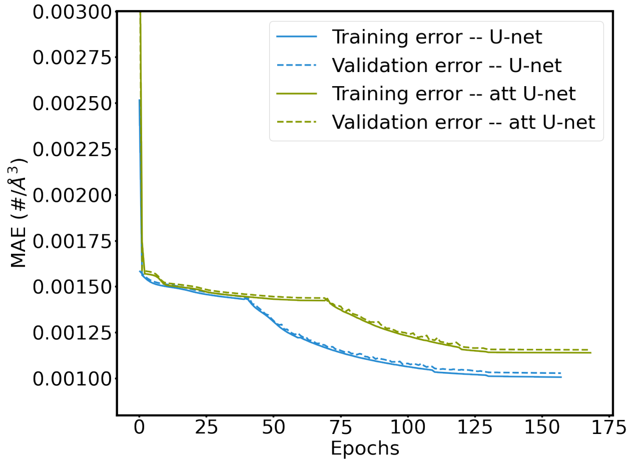


**Fig. S3** The errors during the training of the U-net and the attention U-net over the calcite database. A step scheduler is used to control the learning rate, and hence, the errors drops drastically at certain epochs.

— is used as the loss function to calculate the gradients for the backward propagation. This is calculated between the predicted and the simulated water densities, $\Theta(x)$ and $y$ respectively, given as:

$$L = \sum_{i,j,k=0}^{n_i,n_j,n_k} \frac{|y_{i,j,k} - \Theta(x)_{i,j,k}|}{n_i * n_j * n_k} \tag{5}$$

We trained these models on a NVIDIA® Tesla® V100 GPU, with a 6-core CPU for loading data from the storage.

## Supplemental figures

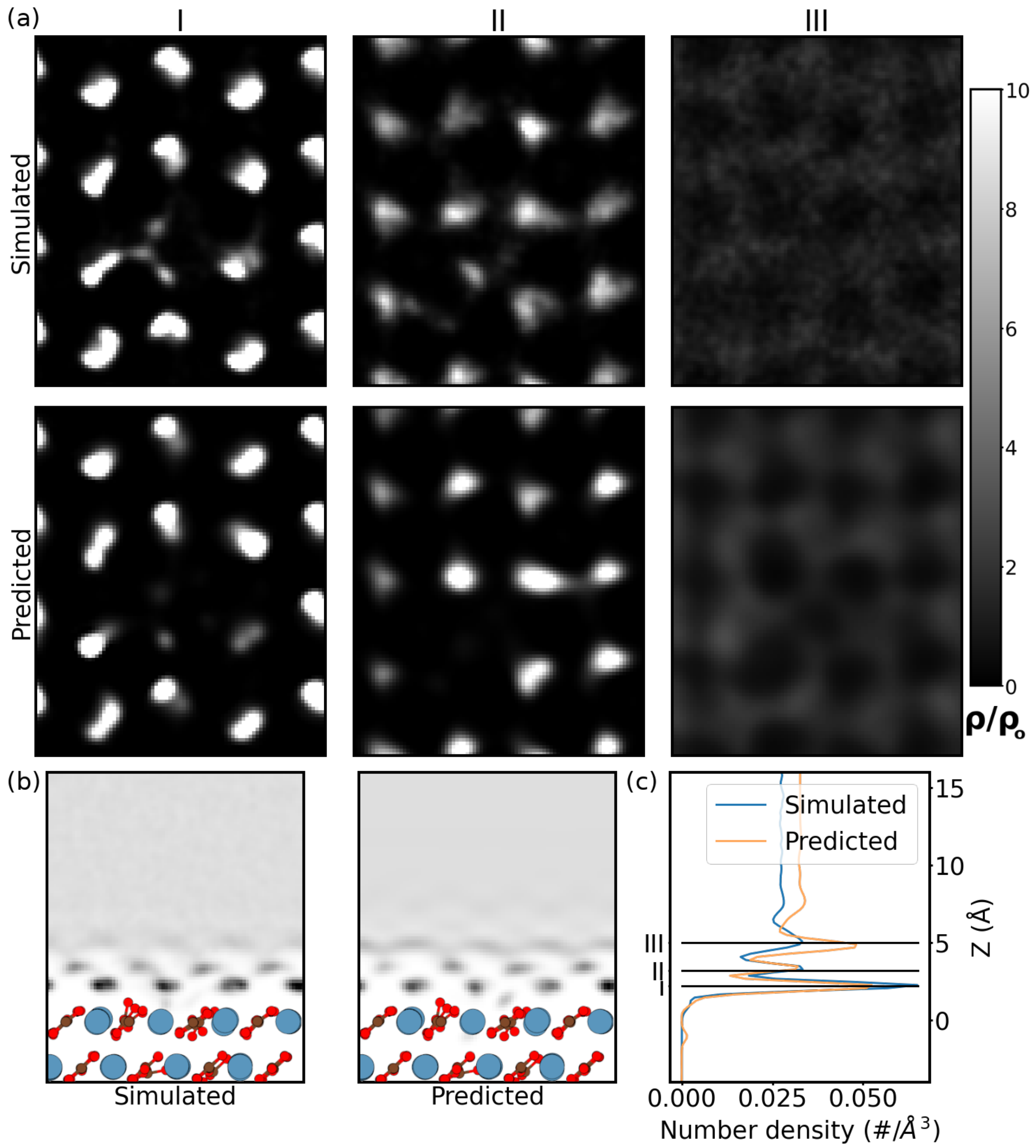


**Fig. S4** Prediction of hydration layers, over a surface with $Ca^{2+}$ vacancy, using the U-Net. (a) Comparison of 2D slices in simulated and predicted water density at z heights corresponding to the peaks in the simulated data. The density ($\rho$) is scaled with the bulk water density ($\rho_o$) for the 2D slices. (b) the mean water density in the 2D $xz$ plane, (c) the 1D water density along the $z$ direction. In the 2D data, the atoms C, O, and Ca, are represented as circles of brown, blue, and red colour, respectively.

## Notes and references


1 S. A. Markgraf and R. J. Reeder, *American Mineralogist*, **70**, 590–600.

2 E. N. Caspi, B. Pokroy1, P. L. Lee, J. P. Quintana and E. Zolotoyabko, *Acta Crystallographica Section B: Structural Science*, **61**, 129–132.

3 R. Demichelis, P. Raiteri, J. D. Gale and R. Dovesi, *Crystal Growth & Design*, **13**, 2247–2251.

4 H. Söngen, B. Reischl, K. Miyata, R. Bechstein, P. Raiteri, A. L. Rohl, J. D. Gale, T. Fukuma and A. Kühnle, *Physical Review Letters*, 2018, **120**, year.

5 J. Tracey, K. Miyazawa, P. Spijker, K. Miyata, B. Reischl, F. F. Canova, A. L. Rohl, T. Fukuma and A. S. Foster, *Nanotechnology*, **27**, 415709.

6 T. Fukuma and R. Garcia, *ACS Nano*, 2018, **12**, 11785–11797.

7 R. W. Hockney and J. W. Eastwood, *Computer simulation using particles*, A. Hilger, Bristol [England] ; Philadelphia, Special student ed edn, 1988.

8 K. Momma and F. Izumi, *Journal of applied crystallography*, **44**, 1272–1276.

9 D. P. Kingma and M. Welling, *arXiv:1312.6114 [cs, stat]*, 2013.

10 A. L. Maas, A. Y. Hannun and A. Y. Ng, *Proc. ICML*, **30**, 3.

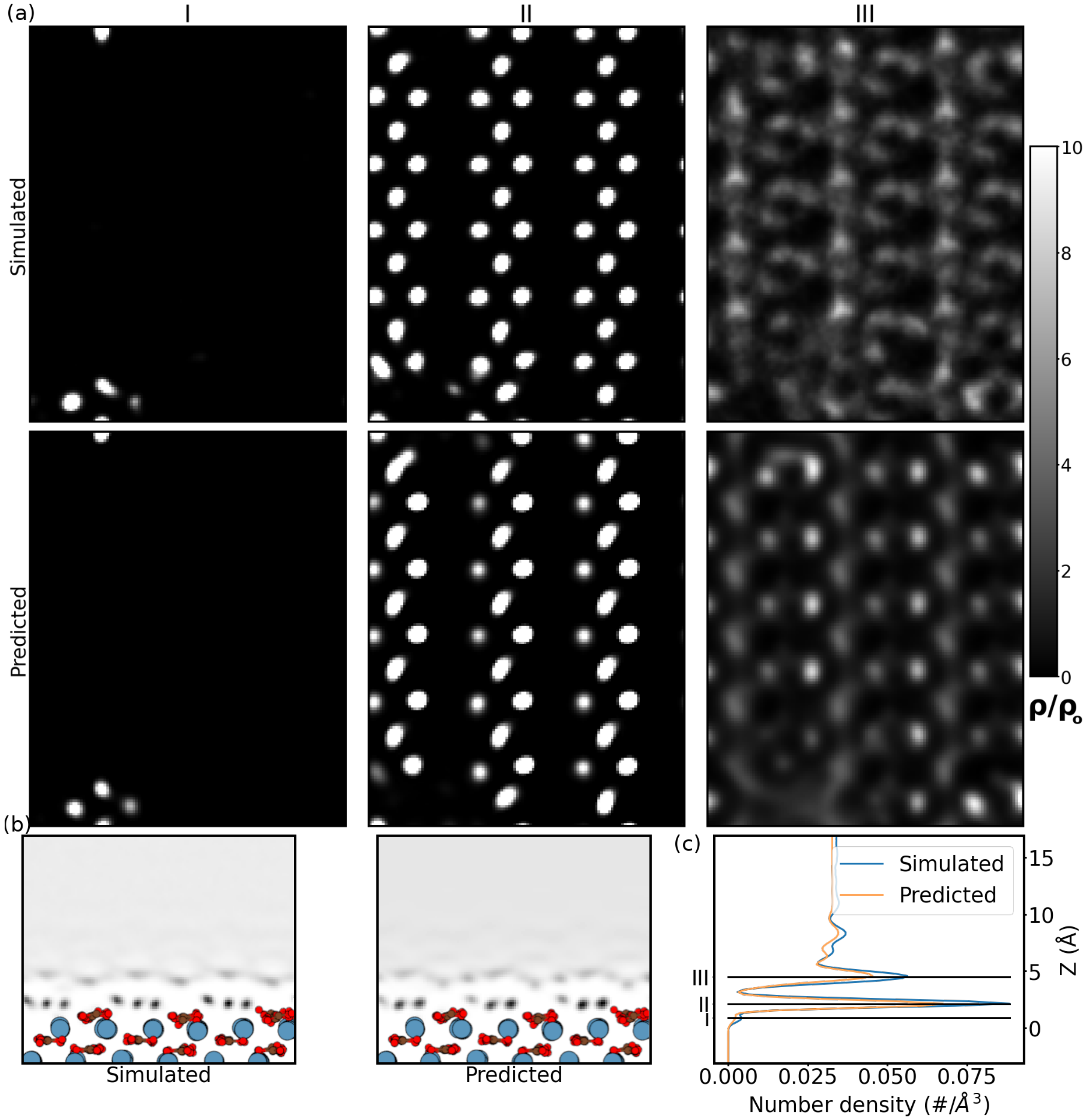


**Fig. S5** Prediction of hydration layers over (001) aragonite surface using the U-Net. The MAE of prediction is 1.1669e-02 $\#/\text{Å}^3$. (a) Comparison of 2D slices in simulated and predicted water density at z heights corresponding to the peaks in the simulated data. The density ($\rho$) is scaled with the bulk water density ($\rho_o$) for the 2D slices. (b) the mean water density in the 2D $xz$ plane, (c) the 1D water density along the $z$ direction.


11 D. E. Rumelhart, G. E. Hintont and R. J. Williams, *Nature*, **323**, 533–536.

12 O. Oktay, J. Schlemper, L. L. Folgoc, M. Lee, M. Heinrich, K. Misawa, K. Mori, S. McDonagh, N. Y. Hammerla, B. Kainz, B. Glocker and D. Rueckert, *arXiv:1804.03999 [cs]*.

13 M. D. Zeiler, *arXiv preprint arXiv:1212.5701*.
